# Prevalence and effects of menstrual disorders on quality of life of female undergraduate students in Makerere University College of health sciences, a cross sectional survey

**DOI:** 10.1186/s12905-023-02290-7

**Published:** 2023-03-30

**Authors:** Emmanuel Odongo, Josaphat Byamugisha, Judith Ajeani, John Mukisa

**Affiliations:** 1 Department of Obstetrics and Gynecology, Makerere University, Kampala, Uganda; 2 Department of Obstetrics and Gynecology, Mulago Specialized Women’s and Neonatal Hospital, Kampala, Uganda; 3 Department of Immunology and Molecular Biology, Makerere University, Kampala, Uganda

**Keywords:** Menstrual disorders, Prevalence, Quality of life, Undergraduate student

## Abstract

**Background:**

Menstrual problems affect as high as 90% of adolescent females and are the main reason for gynecology visits. Dysmenorrhea was the most frequent menstrual disorder for which adolescents and their parents were referred to a physician. Many undergraduate students are adolescents who undergo several hormonal changes that affect menstrual patterns. This study aimed to determine the prevalence of menstrual disorders and to determine the effect of menstrual disorders on quality of life (QOL) of female undergraduate students at Makerere University college of health sciences.

**Methods:**

A cross sectional study design using a self-administered questionnaire. WHO (World Health Organization) QOL-BREF (QOL-Best Available Reference document) questionnaire was used to assess QOL of participants. Collected data was double entered into EPIDATA, and transferred to STATA for analysis. Data was presented using tables, and analyzed using percentages, frequencies, medians, interquartile range, means and standard deviations, t-test and ANOVA were used to establish statistical significance. *P* < 0.05 was considered statistically significant.

**Results:**

Of the participants, 275 were included in the data analysis. The median age of the participants was 21 years with range of 18–39 years and interquartile range of 20–24 years. All the participants had attained menarche. Of the participants, 97.8% (95%CI: 95.2–99.0) (269/275) reported some form of menstrual disorder. Premenstrual symptoms were the commonest disorder (93.8% (95%CI: 90.2–96.1), *N* = 258) followed by dysmenorrhea (63.6% (95% CI: 57.7–69.1), *N* = 175), irregular menstruation (20.7% (95%CI: 16.3–25.9), *N* = 57), frequent menstruation (7.3% 95% CI:4.7–11.0, *N* = 20) and infrequent menstruation (3.3% (95% CI:1.7–6.2), *N* = 9). Dysmenorrhea and premenstrual symptoms significantly reduced the QOL scores of participants.

**Conclusion:**

Menstrual disorders were highly prevalent with negative effects on QOL and class attendance. Efforts should be made to screen and possibly treat menstrual disorders among university students as well as to conduct further studies to elucidate more on the effects of menstrual disorders on quality of life.

**Supplementary Information:**

The online version contains supplementary material available at 10.1186/s12905-023-02290-7.

## Introduction

Menstrual disorders refer to abnormalities of menstruation that include menstrual cycle irregularities; irregular menstrual cycle, heavy menstrual bleeding, infrequent menstruation, frequent menstruation, intermenstrual bleeding, amenorrhea [1], dysmenorrhea, and premenstrual syndrome [2]. Menstrual problems affect 75% of adolescent females and are the main reason for gynecology visits [3]. Dysmenorrhea was reported as the most frequent menstrual disorder for which adolescents and their parents were referred to a physician [4] and a leading cause for long term school absenteeism [5] in the USA. A study in USA reported 90% prevalence of dysmenorrhea [6] and this makes it a public health concern. It has been reported that 10% of women with dysmenorrhea suffer severely enough to render them incapacitated for 1–3 days each menstrual cycle and this affects their QOL, personal health and has a global economic impact [7]. In addition, some girls are unaware that their bleeding patterns are abnormal and can lead to significant long-term health consequences.

Many undergraduate students are adolescents who undergo several hormonal changes that affect menstrual patterns. Menstrual disorders can be confused with menstrual pattern variation caused by these hormonal changes. This variability in menstrual patterns poses a diagnostic challenge to clinicians and may lead to delay of treatment of underlying conditions [8]. Investigations in various developing countries show that women are concerned by menstrual disorders. It has been reported that data in low and middle income countries on the frequency of menstrual disorders and their impact on women’s health status, quality of life and social integration suggest that evaluation and treatment of menstrual complaints should be given a higher priority in primary care programs [9].

In a study of the prevalence and patterns of menstrual symptoms among Lebanese nursing students, the most common menstrual disorders were irregular frequency of menstruation (80.7%), premenstrual syndrome (54.0%), irregular duration of menstruation (43.8%), dysmenorrhea (38.1%), polymenorrhoea (37.5%) and oligomenorrhoea (19.3%) [10].

Whereas most studies in Uganda focused on menstrual hygiene management, one study reported that 19.7% of participants from selected secondary schools in Entebbe missed school at least once during their most recent menstrual period and those who used a menstrual diary reported 28% school absenteeism on period days compared to 7% on non-period days and that missing school during the most recent period was associated with physical symptoms; headache, stomach pain, back pain and with changing protection 4 or more times per 24 h period [11]. This study however did not include the effect of menstrual disorders on quality of life. School absenteeism has been shown to lead to poor scores [12]. A study among university students in Kampala reported a 75.8% prevalence of dysmenorrhea [13] while another study in secondary school students reported a majority of participants with menstrual anxiety [14].

This study aimed at determining the prevalence of menstrual disorders and how they impact on Quality of life of undergraduate students at Makerere University College of health sciences and generate information to foster further research as well as policy on menstrual management.

## Methods

### Study design

A cross sectional study.

### Study setting and study population

The study was conducted in Makerere University, the largest public University in Uganda located about 2 km north of Kampala City with a student population of 39,546, 44% (17,400) being females as of 2017, specifically in Makerere University College of health sciences. The college of health sciences has school of medicine, school of biomedical sciences, school of health sciences and the school of public health, and has a diverse population of students from all over the country as well as international students that makes it a representative sample population. Female undergraduate students who consented to the study were included while those on hormonal contraception or those who were pregnant or lactating were excluded from the study.

### Sampling techniques and procedure

The sample size obtained using the Kish Leslie (1965) formula basing on a previous study in Nigeria by Amu & Bamidele, 2014 was 265, assuming withdrawal of consent or incomplete data of 10%, we aimed at a minimum of 265/0.9 = 295, thus a sample size of 295 was used. Study participants were selected by consecutive sampling of available female undergraduate students in Makerere University College of health sciences.

### Data collection tools, procedure and analysis

Data was collected using a pre-tested self-administered questionnaire from a population of female undergraduate students at Makerere University College of health sciences who enrolled for the study having met the eligibility criteria. The questionnaire contained questions on sociodemographic characteristics such as age, course, year of study, number of pregnancies, education sponsor, etc. menstrual characteristics such as presence or absence of menstruation, age at menarche, length of cycles, regularity of cycles, presence or absence of painful menstrual, duration of menstruation, number of sanitary pads used each menstruation to quantify the amount of bleeding, the severity of menstrual pain (mild, moderate, severe), premenstrual symptoms and participants’ perception of menstrual pain based on their experience were collected. Menstrual disorders were defined as follows: Normal menstrual frequency was defined as menstrual cycles repeated about once every 24–38 days, regular menstrual cycle referred to cycles in which the shortest to longest cycle variation is ≤ 9 days, irregular menstrual cycle referred to cycles in which the shortest to longest cycle variation is ≥ 10 days, infrequent menstruation referred to a menstrual cycle repeated about once every > 38 days, frequent menstruation referred to a menstrual cycle repeated once every < 24 days, intermenstrual bleeding referred to bleeding between cyclically regular onset of menses, dysmenorrhea referred to painful menstruation, premenstrual symptoms were symptoms preceding menses and resolving with onset of menses and included painful breast on touching, difficulty concentrating, mood changes and depression, primary amenorrhea referred to no menses by age 16 years, secondary amenorrhea referred to no menses for an interval of time equivalent to a total of at least three months in students who had menstruated previously.

QOL was measured using the WHO QOL BREF (Field trial version) [15] which comprises of 4 domains; physical, psychological, social and environmental. Physical domain measures activities of daily living, dependence on medicinal substances and medical aids, energy and fatigue, mobility, pain and discomfort, sleep and rest, and work capacity; psychological domain measures bodily image and appearance, negative feelings, positive feelings, self-esteem, spirituality/religion/personal beliefs, thinking, learning, memory, and concentration; social domain measures personal relationships, social support, and sexual activity; environmental domain measures financial resources, freedom, physical safety and security, health and social care: accessibility and quality, home environment, opportunities for acquiring new information and skills, participation in and opportunities for recreation/leisure activities, physical environment, and transport. The scores for each domain being scaled in a positive direction from 0–100.

The WHOQOL-BREF [15] produced a quality of life profile from which four domain scores were derived. The four domain scores that denote an individual’s perception of quality of life in each particular domain were scaled in a positive direction (i.e., higher scores denote higher quality of life). The mean score of items within each domain was used to calculate the domain score. Mean scores were then multiplied by 4 in order to make domain scores comparable with the scores used in the WHOQOL -100. The first transformation method converted the domain scores to range between 4–20, comparable with the WHOQOL-100. The second transformation method converted domain scores to a 0–100 scale. Where more than 20% of data was missing from an assessment, the assessment was discarded. Where an item was missing, the mean of other items in the domain was substituted. Where more than two items were missing from the domain, the domain score was not calculated (with the exception of domain 3, where the domain would only be calculated if ≤ 1 item is missing).

Pretesting questionnaire was done by collecting data from a set of 30 students who were not part of the final study participants and the questionnaire was found to be appropriate. The WHO QOL BREF has been validated in multiple studies as a tool for measuring QOL [16, 17] and we used it without any modification in this study and this part of the questionnaire was not pretested. Participants were physically approached by the study team immediately after classes/lectures/ward rounds during normal working hours on working days and briefed about the study. Eligible students who were willing to participate were given consent forms to fill. Research assistants gave pretested questionnaires to all eligible undergraduate students in Makerere University College of health sciences who accepted and consented to the study. The filled questionnaires were collected on the same day, assembled at a designated point and cross-checked for any errors. The participants were accorded privacy while entering their information.

The research assistants were trained on the research protocol, ethical issues and data collection procedures. Completed questionnaires were double-checked for completeness and accuracy and stored under key and lock by the principal investigator who was also the only person with permission to access the data for confidentiality purposes. There was double entry of data into EPIDATA manager version 4.6.0 and the two entries compared and contrasted to eliminate errors in data entry. Data cleaning to ensure consistency and data integrity was done before export to STATA version 14.1 (Stata Corp, college Station, Texas, USA).

Participant characteristics such as age, menarche, were described using percentages, frequencies, tables, medians and their interquartile range. The prevalence of menstrual disorders was calculated and presented as proportions. The domains of the QOL BREF were summarized using means and 95% confidence intervals because the data were normally distributed. ANOVA test and t-test were used to establish the relationship between the reported menstrual characteristics and the mean domain scores in the QOL BREF, *P* < 0.05 was considered statistically significant. The mean domain scores of QOL in participants with menstrual disorders were compared with those without. The strength of the associations between menstrual disorders and QOL was tested using Cohen’s d effect size calculated as the difference in means divided by pooled standard deviation. Cohen’s d > 0.25 was considered to indicate practical Independent variables were dysmenorrhea, premenstrual symptoms, heavy menstrual flow, amenorrhea, frequent menstruation, irregular menstruation, intermenstrual bleeding, infrequent menstruation while the dependent variables were the quality-of-life Domains in the WHO QOL BREF i.e., physical, psychological, social and environmental domains.

### Ethical considerations

Informed consent was obtained from participants and only those who agreed by signing in the consent form were recruited in the study. Protocol was handed to the department of obstetrics and gynecology Makerere University for review after which it was forwarded to the Makerere University School of medicine research and ethics committee (SOMREC) for approval (approval number: REC REF 2020–122). Copies of permission letters and attached copies of the protocol were distributed to the principle of the Makerere University College of health sciences seeking his permission to collect data. Participation in the study was completely voluntary and participants were free to withdraw from the study any time they wished. Information obtained from the participants were kept confidential and anonymous by assigning code numbers for participants’ questionnaires that were used on all research notes and documents and keeping questionnaires and any other identifying participant information in a locked file cabinet in the personal possession of the researcher.

## Results

### Demographic characteristics of the study participants

Participant response rate was 96% (295/306). Table 1 shows the sociodemographic characteristics of the participants. A total of 295 female students from the college of health sciences returned the questionnaires, 20 of whom had incomplete or missing data and were excluded from the data analysis leaving 275 participants in the data analysis. The median age of the participants was 21 years with range of 18–39 years and interquartile range of 20–24 years.

**Table 1:** Sociodemographic characteristics of the study participants

Characteristic	Frequency (*n* = 275)	Percentage
**Age in years**		
18–19	31	11.3
20–24	204	74.2
25–29	29	10.5
30–39	11	4.0
**Year of study**		
One	89	32.4
Two	86	31.2
Three	46	16.7
Four	20	7.3
Five	34	12.4
**Course/program of study**		
MBChB	152	55.3
Biomedical Sciences	28	10.2
Nursing	33	12.0
Pharmacy	16	5.8
Optometry	9	3.3
Cytotechnology/biomedical engineering	11	4.0
BDS	11	4.0
BMR/BSLT	15	5.5
**Number of pregnancies**		
0	250	90.9
1	16	5.8
2	7	2.5
3	2	0.7
**Education Sponsor**		
Parent	142	51.6
Guardian	44	16.0
Other (government, NGO, Project, self)	89	32.4

### Prevalence of menstrual disorders

The mean age of menarche was 13.07 ± 1.37 years. Table 2 shows the menstrual characteristics of the participants. Of the participants, 97.8% (95% CI: 95.2–99.0) (269/275) reported at least one form of menstrual disorder, 6.3% (95% CI: 3.9–9.9) and 11.2% (95%CI: 7.89–15.5 had early menarche (< 12 years) and delayed menarche (> 14 years) respectively.

**Table 2:** Menstrual characteristics of participants

Characteristic	Frequency	Percentage and 95% CI
**Age at menarche (** ***n*** ** = 269)**		
< 12	17	6.3 (3.9–9.9)
12–14	222	82.5 (77.5–86.6)
15–17	30	11.2 (7.9–15.5)
**Regular menstrual periods (inter- cycle difference ≤ 9 days)? (** ***n*** ** = 275)**		
Irregular menstruation	57	20.7 (16.4–25.9)
Regular menstruation	218	79.3 (74.0–83.7)
**Duration of menstrual cycle in days, ** ***n*** ** = 275**		
Normal cycle (24.0–38.0 days)	246	89.4 (85.2–92.5)
Frequent menstruation (< 24.0 days)	20	7.3 (4.7–11.0)
Infrequent menstruation (> 38.0 days)	9	3.3 (1.7–6.2)
**Intermenstrual bleeding**		
No	254	92.4 (88.5–94.9)
Yes	21	7.6 (5.01–11.5)
**Dysmenorrhea (painful periods)**		
No	100	36.4 (30.8–42.2)
Yes	175	63.6 (57.7–69.1)
**Frequency of menstrual pain (** ***n*** ** = 175)**		
All periods	82	46.8 (39.5–54.3)
Some periods	71	40.6 (33.4–48.1)
Occasionally	22	12.6 (8.4–18.4)
**Pain severity (** ***n*** ** = 175)**		
Mild	34	19.4 (14.1–26.0)
Moderate	98	56.0 (28.5–63.2)
Severe	43	24.6 (18.70–31.6)
**Premenstrual symptoms**		
Painful breast on touching	111	40.4 (34.7–46.3)
Difficulty concentrating	56	20.4 (15.9–25.6)
Depression/ Mood changes	167	60.7 (54.8–66.3)
**Presence of premenstrual syndrome symptoms**		
No	17	6.2 (3.9–9.7)
Yes	258	93.8 (90.2–96.1)
**Consultations because of menstrual problems** ^a^		
Doctor	38	13.8
School nurse	21	7.6
Parent	72	26.2
Friend	66	24.0
None	142	51.6
**Missed class (es) in the last 2 months because of a menstrual problem**		
No	245	89.1
Yes	30	10.9
**What is your perception about menstrual pain**		
Normal occurrence, yes	152	55.3
Bad occurrence, tolerable, yes	87	31.6
Bad occurrence requires treatment	28	10.2
Missing	8	2.9
**Do you think your periods are normal?**		
No	12	4.4
Yes	193	70.2
Unsure	70	25.4
**Had at least one disorder**		
No	6	2.2
Yes	269	97.8

^a^Participants had multiple experiences

### Menstrual disorders and quality of life

Table 3 shows the proportion of the participants with menstrual disorders who missed classes. Tables 4 and 5 show the effect of menstrual disorders on WHO QOL BREF domain scores. Most of participants who reported any of the menstrual disorders scored less in quality of life compared to those who did not though in some instances the differences were not statistically significant.

**Table 3:** Distribution of students with menstrual disorders who reported missing classes in the 2 months preceding the study

Characteristic	Missed classes, N(%), *n* = 30	95% Confidence interval
Intermenstrual bleeding	4 (13.3)	4.9–31.1
Dysmenorrhea	30 (100.0)	
Frequent menstruation	2 (6.7)	1.6–23.6
Infrequent menstruation	1 (3.3)	0.4–20.9
Irregular menses	5 (16.7)	6.9–34.8
Premenstrual syndrome	28 (93.3)	76.4–98.3

**Table 4:** Effects of dysmenorrhea, pre-menstrual syndrome symptoms and intermenstrual bleeding on mean QOL BREF domain scores

QOL BREF DOMAINS	Dysmenorrhea			Premenstrual symptoms			Intermenstrual bleeding		
	No (mean QOL score,95%CI)	yes, (mean QOL score,95%CI, Effect size)	*P* value^*^	No (mean QOL score,95%CI)	Yes (mean QOL score,95%CI, Effect size)	*P* value^*^	No (mean QOL score,95%CI)	Yes (mean QOL score,95% CI, Effect size)	*P* value^*^
Physical	62.8, 60.4–65.2	62.0, 60.1–65.9, 0.065	0.601^*^	60.9,54.5–67.3	62.4, 60.9–63.9, 0.120	0.623^*^	62.1,60.6–63.7	64.5,59.2–69.9, 0.199	0.395^*^
**Psychological**	**60.4, 57.9–62.9**	**56.2, 55.1–57.2, 0.377**	** < 0.001** ^*^	62.0, 56.3–67.7	57.4,56.2–58.6, 0.447	0.057^*^	57.8, 56.6–58.9	56.6, 51.4–61.8, 0.115	0.587^*^
**Social**	**59.7,57.1–62.3**	**51.6, 50.2–53.1, 0.696**	** < 0.001** ^*^	56.9, 48.4–65.3	50.4, 53.0–55.8, 0.581	0.404^*^	54.8, 53.4–56.3	51.4,45.7–57.2, 0.280	0.204^*^
**Environmental**	**50.3, 49.2–51.5**	**45.9, 44.2–47.5, 0.504**	** < 0.001** ^*^	**58.8, 53.3–64.4**	**46.7, 45.6–47.8, 1.212**	** < 0.001**	47.7, 46.5–48.9	44.9, 39.5–50.3, 0.2611	0. 201^*^

^**^ *P* value based on one-way analysis of variance (ANOVA)

^*^ *P* value based on t-test

**Table 5:** Effects of frequent menstruation, infrequent menstruation and irregular menstrual cycle on QOL domain scores

QOL BREF DOMAINS	Menstrual frequency				Regular menstrual cycle		
	Frequent menstruation (mean QOL score, 95% CI, Effect size)	Normal menstrual cycle (mean QOL score, 95% CI,)	Infrequent menstruation (mean QOL score 95% CI, Effect size)	*P* value^**^	No (mean QOL score, 95% CI, Effect size)	Yes (mean QOL score, 95% CI)	*P* value^*^
Physical	63.9, 62.0–65.8, 0.1343	62.3,62.2–62.4	58.3, 56.2–60.4, 0.3136	0.534^**^	60.4,56.9–63.9, 0.188	62.8, 61.2–64.4	0. 195^*^
Psychological	58.0, 56.7–59.3, 0.0232	57.8, 57.7–57.9	54.7, 52.7–56.7, 0.277	0.624^**^	56.0, 53.2–58.8, 0.219	58.2, 56.9 -59.4	0.129^*^
Social	50.8, 48.4–53.2, 0.2786	54.7, 54.6–54.8	59.0, 57.6–60.4, 0.365	0.181^**^	52.1, 48.3–55.8, 0.246	55.2, 53.7–56.7	0.072^*^
Environmental	49.1, 47.6–50.6, 0.1952	47.3, 47.2–47.4	48.4,47.1–49.7, 0.121	0.699^**^	48.6, 46.0–51.1, 0.146	47.2,45.9–48.5	0.339^*^

^**^ *P* value based on one-way analysis of variance (ANOVA)

^*^ *P* value based on t-test

## Discussion

Menstruation is a normal physiologic process but is frequently complicated by pre-menstrual and menstrual disturbances [18]. Disorders of menstruation could be manifestations of serous underlying health problems that if not detected can progress causing significant morbidity. School absenteeism and interference with life activities/quality of life are some of the reported consequences of menstrual disorders [19].

### Prevalence of menstrual disorders

In this study we established the prevalence of menstrual disorders and their effect on the quality of life of female undergraduate students in Makerere University college of health sciences. The participants comprised mainly young people. Of the 275 participants, 269 (97.8%, 95%CI: 95.2–99.0) reported one of the different forms of menstrual disorders considered in this study including irregular menstrual cycle, frequent menstruation, infrequent menstruation, intermenstrual bleeding, premenstrual symptoms and dysmenorrhea. A similar high prevalence of 91% was reported among students studying health sciences in Saudi Arabia [20]. It is worth noting that in spite of the high prevalence of menstrual disorders (97.8%), only 4.4% (12/275) perceived their menstruation as abnormal indicating that university students are not aware of what constitutes a normal menstruation verses abnormal menstruation and thus may suffer from complications due to delayed diagnosis of underlying conditions if any. More worrying is that 55.3% of participants reported that menstrual pain is a normal occurrence while only 13.8% of participants reported consulting a medical doctor for a menstrual problem denoting very poor health seeking behavior for menstrual problems. Participants who reported menstrual disorders had lower quality of life scores in most domains compared to their counterparts without such disorders though in some cases the differences were not statistically significant. 10.9% (30/275) of participants reported missing classes in the preceding 2 months before the study. 100% of participants who missed classes had dysmenorrhea while 93.8% had at least one feature of premenstrual syndrome indicating that these two disorders are the leading causes of missed classes in this study population.

The prevalence of dysmenorrhea in this study was 63.6% (95% CI 57.7–69.1) with 46.8% reporting dysmenorrhea in all menses and 40.6% reporting it in some menses. The prevalence of dysmenorrhea in this study is lower than that reported in northern Ghana (83.6%) [21], Spain (74.8%) [22] and the United States of America (85%) [21] and higher than that reported in Hunan, China(41.7%) [23] and Nigeria (53.3%) [24]. Wide variations in prevalence of dysmenorrhea have been reported across the world with one systematic review reporting the prevalence of menstrual pain between 16.38%-90.4% (median = 83.8) and most students reported their pain as moderate [25]. These variations could be due to differences in population characteristics, genetics, race, lifestyle and socioeconomic factors as well as lack of universally accepted definition of dysmenorrhea [21]. 24.6% of respondents in this study reported their pain as severe while 56% reported moderate pain implying that dysmenorrhea is a significant public health problem.

Irregular menstruation defined as menstrual cycle variation of more than 9 days was reported by 57 (20.7%, 95% CI 16.4–25.9) of participants. This prevalence is lower than that reported among university students in Turkey (31.2%) [26]. A Danish study reported menstrual cycle variation of greater than 14 days in 29.3% of participants among both adolescent and adult women [27]. The Danish study however, defined normal cycle length as 21–35 days and involved a larger number of adult women as opposed to this study in which we defined normal menstrual cycle length as 24–38 days and only 14.5% of participants were more than 25 years old. 89.4% of participants in this study reported normal cycle length, 7.3% reported frequent menstruation and 3.3% reported infrequent menstruation. An Indian study reported that 97.2% of medical students had cycle length of 28–35 days [7] which is higher than what we found in this study and only 2.8% of the participants reported menstrual cycle abnormalities unlike the 10.6% in this study. The differences could be explained by the different definitions for normal menstrual cycle length used in the studies.

Of the participants, 93.8% (95% CI 90.2–96.1) reported at least one premenstrual symptom. The most prevalent symptoms were depression/mood changes (60.7%) and painful breast on touching (40.4%) This prevalence is higher than the 79% reported among Japanese College students [28] and among young female students studying health sciences in Saudi Arabia (46.7%) [20]. Varying prevalence of premenstrual symptoms have been reported globally, the lowest in France (12%) and the highest in Iran (98%) according to a metanalysis [29]. Variations in the frequency of possible causes of premenstrual symptoms such as malfunction of the hypothalamo-pituitary-axis, nutritional factors, hormonal imbalance and environmental factors [20] in different populations could contribute to the differences in prevalence of premenstrual symptoms as well as varying definitions of premenstrual disorders used in different studies.

None of the participants reported missing menses in the 3 consecutive months preceding the study indicating no cases of primary or secondary amenorrhea.

### Effects of menstrual disorders on quality of life of participants

Dysmenorrhea and premenstrual symptoms had statistically significant negative effects on the quality of life of participants in the environmental (45.9 vs 50.3, *p* < 0.001, effect size d = 0.504), psychological (56.2 vs 60.4, *p* < 0.001, effect size d = 0.377) and social domains (51.6 vs 59.7, *p* < 0.001, effect size d = 0.696), and environmental domain (47.7 vs 58.8, *p* < 0.001, effect size d = 1.212) respectively. These effect sizes demonstrate practically significant associations. 10.9% (30/275) of participants reported ever missing class in the 2 months preceding the study. 100% (30/30) of participants who missed classes had dysmenorrhea while 93.3% had premenstrual symptoms. Intermenstrual bleeding, frequent menstruation, infrequent menstruation and irregular menstruation did not significantly reduce quality of life of the participants.

In India dysmenorrhea reduced the QOL WHO BREF scores in all domains [30] similar to what we found in this study in which dysmenorrhea significantly reduced QOL scores in the psychological, social and environmental domains. The study in India was community based and the participants were older (mean age of 33 years).

Dysmenorrhea was reported to negatively affect the health related quality of life of university students in Western Turkey [31]. This study used the Short-Form-36 for assessing the health-related quality of life unlike in our study in which we used the WHO QOL BREF to assess QOL in entirety. Both studies demonstrate the negative effect of dysmenorrhea on quality of life of university students. The WHO QOL BREF was validated across several countries including Zimbabwe [32] which has comparable characteristics to Uganda. Dysmenorrhea significantly affected the total score for perceived quality of life of Spanish University students using the EuroQol-5 dimensions 5-levels (EQ-5D-5L) [19]. The congruence and convergence of these different QOL assessment tools indeed highlights the fact that dysmenorrhea negatively impacts QOL of University students.

In studying the relationship between premenstrual symptoms, menstrual pain, irregular menstrual cycles, and psychosocial stress among Japanese college students, Yamamoto et al. reported that students who reported dysmenorrhea, irregular menstrual cycles and premenstrual symptoms had higher scores perceived stress than those who did not [28]. Since high stress scores are closely linked to poor QOL scores [33], it can be deduced that premenstrual symptoms, menstrual pain and irregular menstrual cycles negatively impacted the QOL of Japanese college students. However irregular menstrual cycles did not significantly reduce the QOL scores among the Makerere University College of health sciences students. In Saudi Arabia, premenstrual syndrome significantly influenced daily activities related to quality of life and homework of medical students [34]. This study population is comparable to our study population that comprised mostly medical students.

QOL being an individual’s perception of their position in life in the context of culture and value system where they are inserted, which also involves their goals, perspectives, standards and concerns [32] could be blurred during assessment by ignorance about acceptable health standards. This could explain why much as 97.8% of our study participants reported some form of menstrual disorder, there were no significant differences in quality of life domain scores in most domains because the students were not concerned about their menstrual abnormalities with only 4.4% perceiving their menstruation as abnormal.

We believe these study findings provide a reasonable representation of the prevalence of menstrual disorders and their effects on quality of life of female undergraduate students studying health sciences in Uganda, however more studies with emphasis on the effect of confounders and effect modifiers of QOL in this population would be valuable.

## Limitations of the study

Confounding factors can affect QOL scores other than menstrual disorders [35–37] and we were not able to exclude these in this study, however the statistical significance of the findings and consistency with previous study findings assures of reliability and validity. Participants were required to recall their menstrual experiences and this could lead to recall bias. Diagnosis of menstrual disorders was not done clinically and solely depended on what the participants wrote in the questionnaires. The reported number of sanitary pads used that were used for estimating menstrual blood loss could not have been completely soaked by menstrual blood. Female students who could have missed classes due to menstrual problems might not have been captured. The WHO QOL BREF was validated in only one African country and was not validated in Uganda but we assumed the settings were comparable.

## Conclusions and recommendations

Menstrual disorders were highly prevalent among female students in the college of health sciences. Premenstrual symptoms and dysmenorrhea were the most prevalent menstrual disorders and had significantly negative effects on quality of life of the affected students.

Efforts should be made to screen and possibly treat menstrual disorders among University students to ameliorate their negative effects on quality of life of the affected students. Further studies are necessary to elucidate more on the effects of menstrual disorders on quality of life.

## Supplementary Information

**Additional file 1.**

## Data Availability

All data generated or analyzed during this study are included in this published article [and its supplementary information files].

## Abbreviations

QOL: Quality of Life
PMS: Premenstrual syndrome
USE: Universal secondary education
SOMREC: Makerere University School of medicine research and ethics committee
WHO: World Health Organization
BREF: Best Available Reference document
